# A systematic review of human and animal leptospirosis in the Pacific Islands reveals pathogen and reservoir diversity

**DOI:** 10.1371/journal.pntd.0006503

**Published:** 2018-05-14

**Authors:** Vanina Guernier, Cyrille Goarant, Jackie Benschop, Colleen L. Lau

**Affiliations:** 1 Australian Institute of Tropical Health and Medicine, James Cook University, Townsville, Australia; 2 Global Leptospirosis Environmental Action Network (GLEAN), World Health Organization, Geneva, Switzerland; 3 Institut Pasteur in New Caledonia, Institut Pasteur International Network, Nouméa, New Caledonia; 4 School of Veterinary Science, Massey University, Palmerston North, New Zealand; 5 Research School of Population Health, The Australian National University, Canberra, Australia; Weill Cornell Medical College, UNITED STATES

## Abstract

**Background:**

The Pacific Islands have environmental conditions highly favourable for transmission of leptospirosis, a neglected zoonosis with highest incidence in the tropics, and Oceania in particular. Recent reports confirm the emergence and outbreaks of leptospirosis in the Pacific Islands, but the epidemiology and drivers of transmission of human and animal leptospirosis are poorly documented, especially in the more isolated and less developed islands.

**Methodology/Principal findings:**

We conducted a systematic review of human and animal leptospirosis within 25 Pacific Islands (PIs) in Polynesia, Melanesia, Micronesia, as well as Easter Island and Hawaii. We performed a literature search using four international databases for articles published between January 1947 and June 2017. We further included grey literature available on the internet. We identified 148 studies describing leptospirosis epidemiology, but the number of studies varied significantly between PIs. No data were available from four PIs. Human leptospirosis has been reported from 13 PIs, with 63% of all studies conducted in Hawaii, French Polynesia and New Caledonia. Animal leptospirosis has been investigated in 19 PIs and from 14 host species, mainly pigs (18% of studies), cattle (16%) and dogs (11%). Only 13 studies provided information on both human and animal leptospirosis from the same location. Serology results were highly diverse in the region, both in humans and animals.

**Conclusions/Significance:**

Our study suggests that, as in other tropical regions, leptospirosis is widespread in the PIs while showing some epidemiological heterogeneity. Data are scarce or absent from many PIs. Rodents, cattle, pigs and dogs are all likely to be important carriers, but the relative importance of each animal species in human infection needs to be clarified. Epidemiological surveys with appropriate sampling design, pathogen typing and data analysis are needed to improve our understanding of transmission patterns and to develop effective intervention strategies.

## Author summary

Leptospirosis is an important bacterial zoonosis that affects people and animals worldwide. It is common in tropical areas, especially in island ecosystems. Because islands are relatively small, isolated, and have limited health and diagnostic facilities, the disease burden is often underestimated. In this systematic review, we aimed to describe the extent of leptospirosis in the Pacific Islands, including the diversity of pathogens and animal reservoirs. We identified 148 studies from 21 Pacific islands that described *Leptospira* infection in humans or animals. In hospitalized febrile patients, leptospirosis was a common cause of the acute febrile illness, but accurate diagnosis was challenging and often delayed because symptoms overlapped with many other infectious diseases, and access to laboratory diagnosis was limited. A wide variety of animal hosts of *Leptospira* were identified, with rodents, cattle, pigs and dogs reported as important hosts; however, their relative importance in human infection remains unclear. Our review demonstrates that the epidemiology of leptospirosis varies across the Pacific Islands, but information about risk factors and transmission routes is currently limited. We recommend more integrated studies, using an eco-epidemiological approach that includes human, veterinary and environmental factors, and interactions between factors at different ecological scales.

## Introduction

*Leptospira* is a genus of bacteria belonging to the phylum of Spirochaetes causing leptospirosis in humans and other mammals [1]. Leptospirosis is the most widespread and potentially fatal bacterial zoonosis worldwide [2], with an estimated 1.03 million human cases and 58,900 deaths worldwide each year [3]. The majority of the disease burden occurs in tropical regions where large epidemics can occur after heavy rainfall and flooding [4]. Leptospirosis is a neglected disease in most of the tropics, especially in the Pacific region [5], and a recent systematic review found that Oceania was the region most impacted by leptospirosis in terms of morbidity (150.68 cases per 100,000 per year), mortality (9.61 deaths per 100,000 per year) [3], and disability adjusted life years (DALY) [6]. Incidence of up to 1,945 cases per 100,000 population has been reported in 2008 in Futuna (a Polynesian island) during a multi-year outbreak [7]. The health impacts of leptospirosis have been predominantly attributed to acute infections and early complications such as pulmonary haemorrhage and renal failure. However, leptospirosis can also cause subacute and chronic complications and long-term sequelae [8].

Two classification schemes are used for *Leptospira*, one based on serology with the serovar as the basic taxon, and another which uses molecular taxonomy to identify the species, sometimes referred to as genomospecies [2]. *Leptospira* have been classified serologically into over 300 serovars grouped in almost 30 serogroups (both saprophytic and pathogenic) using Microscopic Agglutination Test (MAT) and Cross Agglutination Absorption Test (CAAT) [9, 10]. Phylogenetically, the genus *Leptospira* is divided into 23 species based on 16S rRNA phylogeny and DNA-DNA hybridization, and clustered into saprophytic, intermediate and pathogenic groups [11]. Laboratory diagnosis of leptospirosis may be accomplished by direct detection of the organism or its components in body fluid or tissues, by isolation of leptospires in cultures, or by detection of specific antibodies [12, 13]. Molecular diagnosis is based on *Leptospira* DNA amplification from serum, urine, aqueous humour, cerebrospinal fluid (CSF) or post-mortem tissue samples [14]. Historically, most cases of leptospirosis have been diagnosed by serology, because capacity for culture and PCR were limited. IgM antibodies are detectable in the blood from 5–7 days after the onset of symptoms [2]. The use of agglutination tests was described soon after the first isolation of the organism, and the MAT remains the definitive serological investigation technique in both humans and animals [15].

Human infections range from, most commonly, a mild ‘flu-like illness, to severe complications including acute renal failure and pulmonary haemorrhagic syndrome associated with high fatality rates. Infection results from direct or indirect exposure to urine from infected reservoir host animals that carry the pathogen in their renal tubules and shed pathogenic leptospires which contaminate soils, surface waters, streams and rivers [2]. Humans are infected via mucous membranes, abrasions or cuts in the skin. Prolonged immersion in, or swallowing of, contaminated water can also result in infection. Numerous animal species, including rodents (often considered as the main reservoir), domestic mammals (including livestock and companion animals) and wildlife, have been shown to be reservoirs for *Leptospira* [15]. In food-producing animals, cattle and pigs are relatively susceptible to clinical infection, resulting in production losses including reduced milk yield, reproductive failure, abortions, premature birth or stillbirth [16].

The Oceania region includes Australia, New Zealand and the Pacific Islands Countries and Territories (PICTs), these latter all falling within the tropics. Poverty, remoteness and tropical climate all contribute to vulnerability to, and significant burden of, infectious diseases in the PICTs [17]. Global emergence of leptospirosis has been associated with environmental factors including rainfall, flooding, poverty, and urbanization [18–20], all of which are important drivers of transmission in the Pacific Islands. Recent reports confirm the emergence of leptospirosis in the Pacific region, with increase in incidence and reports of unprecedented outbreaks [5, 21]. However, the incidence of leptospirosis is unfortunately not well-documented from many Pacific Islands, mainly because of the unavailability of laboratory diagnosis [22, 23], poor medical awareness, and non-specific symptoms that overlap with many other tropical infectious diseases, especially arbovirus infections [24]. As a consequence, little is known about the ecological, epidemiological and clinical characteristics of leptospirosis in the region, and the burden of the disease might be even higher than recognized.

To tackle these gaps in current knowledge and understanding of human and animal leptospirosis infection in the Pacific Islands, we conducted a systematic review of both peer-reviewed and grey literature following the Preferred Reporting Items for Systematic Reviews and Meta-analyses (PRISMA) guidelines [25]. Our aims were to summarise and compare human and animal leptospirosis within and between the Pacific Islands.

## Methods

### Geographic scope

**Fig 1:**
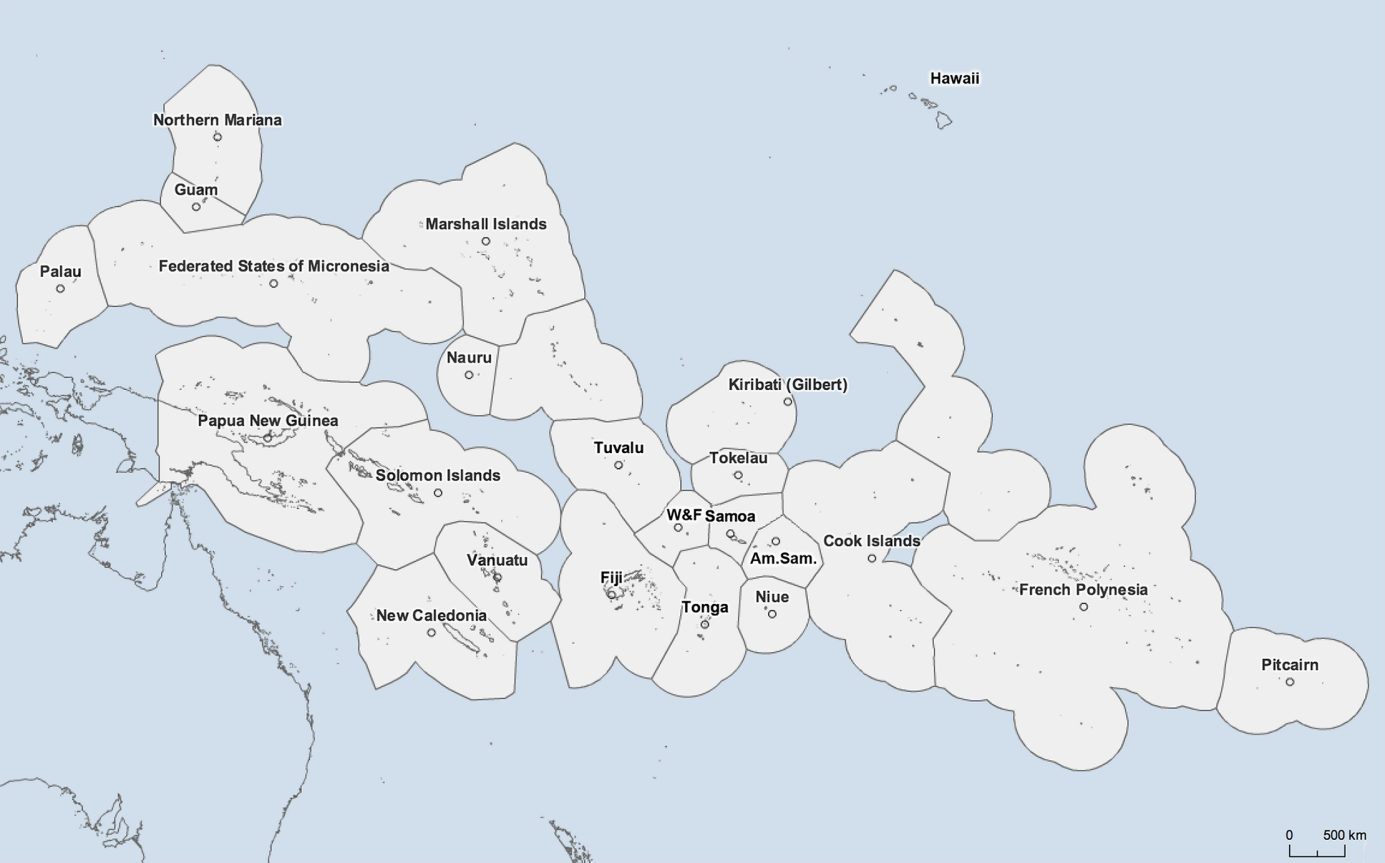
Map of the South Pacific region. All countries in Oceania were included in this review with the exception of Australia and New Zealand. Easter Island does not appear on the map but was included in our study. ‘W&F’: Wallis and Futuna. ‘Am.Sam.’: American Samoa. The map was provided by the Pacific Community (SPC) and is available online at: http://pacific.popgis.spc.int.

The selection of the countries and territories to be included in this review was based on the official list of the United Nations Statistics Division for the geographical region #009 Oceania (http://unstats.un.org/unsd/methods/m49/m49regin.htm#oceania). We excluded Australia and New Zealand (#053) and focused on the small islands of the PICTs including five in Melanesia (Fiji, New Caledonia, Papua New Guinea, Solomon Islands, Vanuatu), eight in Micronesia (Guam, Kiribati, Marshall Islands, Federated States of Micronesia, Nauru, Mariana Islands, Palau, United States Minor Outlying Islands) and ten in Polynesia (American Samoa, Cook Islands, French Polynesia, Niue, Pitcairn Islands, Samoa, Tokelau, Tonga, Tuvalu, and Wallis and Futuna). The “United States Minor Outlying Islands” were restricted to Wake Island in our study. We further included Easter Island (Chile) as well as Hawaii (U.S.A.) (Fig 1). Except for Papua New Guinea and Hawaii, the included PIs had populations of less than one million inhabitants and represented significant diversity in economies, geography, culture and living conditions. For ease of reference, the 25 countries and territories included in this review will be referred to as ‘Pacific Islands’ (PIs).

### Search strategy

Peer-reviewed studies were sought in June 2017 from four international databases (PubMed, Web of Science, Scopus, and Embase (Ovid)) for resources published between January 1947 and June 2017 inclusively, either in English or in French, and following the PRISMA guidelines [25] (S1 Appendix). The search strategy is presented in detail in S2 Appendix. Secondly, additional peer-reviewed studies were retrieved by examining the references from the papers identified by the initial electronic search. Thirdly, grey literature (i.e. print and electronic formats that have not been formally published by commercial publishers) were reviewed by scrutinising the websites of government health departments and other relevant administrative bodies of the Pacific Islands (see S2 Appendix for the list of websites browsed), and by bibliography hand searches of relevant articles from Google Scholar (https://scholar.google.com).

### Eligibility criteria

#### Inclusion criteria

A publication was included in this review if it contained qualitative and/or quantitative information on human or/and animal leptospirosis in the PIs, including environmental studies, case reports, outbreak description or epidemiologic surveys. Conference papers were included if they contained relevant information not published in a peer-reviewed journal.

#### Exclusion criteria for title and abstract review

Reviews or summary articles, editorials, letters to the editor, opinions or commentaries without original data were excluded, as well as lay media publications or broadcasts. We further excluded publications focusing on (i) the wrong geographic location, (ii) the wrong agent/disease, (iii) experimental data (*in vitro* or *in vivo* cellular, molecular, biochemical or other studies that did not include naturally occurring cases of leptospirosis in humans or animals), and (iv) descriptions or studies of laboratory methods. The inclusion and exclusion criteria were applied to the title and abstract of all retrieved references. References for which no abstract was available were included in the next stage of full text review.

#### Exclusion criteria during full-text review

The full texts of articles classified as eligible for inclusion were retrieved and assessed against the same exclusion criteria as per abstract review. Any articles where the country was not specified were excluded, as well as references for which the methods or study population were not described in sufficient detail to determine whether the study met inclusion or exclusion criteria. When more than one reference was retrieved for the same study, or when the same data were retrieved under different formats (publications, project reports, conference presentation), only the most comprehensive one was retained. We included reports of returned travellers despite a potential uncertainty around the specific location where infection was acquired, because for some of the Pacific Islands considered, this was the only information/evidence available. Studies for which the full text was not available were excluded except when relevant data (qualitative or quantitative) were available from the abstract or from a later published study.

### Data collection process

The data collection process was undertaken in two steps. First, abstracts and titles were compiled in EndNote (Thomson Reuters, Philadelphia, PA, USA) and reviewed by one researcher (VG) on the basis of the abstract and title. Second, the articles identified through the pre-selection process were retrieved in full text format and reviewed independently by two researchers for the full text (VG and either CG, JB or CLL). A third researcher served as a tiebreaker for any discordant decisions.

Following the inclusion/exclusion process, qualitative and quantitative data were extracted from each of the included articles. Papers were classified into human clinical studies, human community-based studies, and animal population studies (no animal clinical studies were identified). For human studies, information was compiled on the country, year(s) of study, study design, target population, inclusion criteria, number enrolled, diagnostic tests used, confirmed leptospirosis cases and risk factors. For animal studies, information was compiled on country, year(s) of study, species investigated, methodology, number of samples, and confirmed positivity for *Leptospira*. When available, data on serology and genetic typing of isolates from humans and animals were also compiled and summarised by country and by animal species.

### Case definition and strength of evidence of results

MAT is a serogroup-specific assay and does not provide reliable information on the infecting serovar [26, 27]; results were therefore summarised at the serogroup level even if studies reported MAT results at the serovar level. Although serogroups are no longer used in the taxonomic classification of serovars, they remain useful for laboratory purposes and epidemiological comparisons.

As the definition of a “positive” serology result differed between studies, we standardised results across the studies by using the following case definitions:

1. For the human clinical studies, a confirmed case was defined based on the recommendations of the WHO LERG report [28] as follows: clinical signs and symptoms consistent with leptospirosis and one or more of the following: (i) positive PCR or isolation by culture; (ii) seroconversion or fourfold increase in MAT titre between acute and convalescent serum samples; (iii) MAT titre ≥ 1:400 in single serum sample. Cases that fitted the confirmed case definition were considered “strong evidence” whereas others were considered “weak evidence”.
2. For the seroprevalence studies (human or animal), any positive MAT result (as defined by the individual studies, from 1:50 upwards) was considered to indicate previous infection. However, opportunistic sampling of animals with less than ten individuals of a species was considered as “weak evidence”.
3. Any result based on *Leptospira* isolates was considered “strong evidence”.

When MAT results were detailed, the serogroup with the highest titre was reported as putatively involved. If equally high titres were reported for more than one serogroup, MAT was considered positive but serogroup results were not reported. If data were not detailed enough to allow interpretation (especially in the case of cross-reactions), serogroup results were not reported.

### Critical assessment of data quality and possible bias

Because of the great heterogeneity in the methodology and the quality of the data between studies, most of the data reported in our review are qualitative rather than quantitative. As detailed in the previous paragraph, methodological quality was assessed for serology studies by comparison to pre-determined case definition criteria to control for heterogeneity in study design and diagnostic methodology. Evidence of *Leptospira* infection was assessed for each study as ‘strong’ or ‘weak’. The sample sizes (number of animal tested, number of clinical cases reported) are reported in our review when necessary, as an indication of the degree of confidence of the results. Exposure and risk factors are reported as per the included studies, and are considered mostly as untested hypotheses. Epidemiological studies providing risk factors based on robust analyses are highlighted.

## Results

### Selected studies

**Fig 2:**
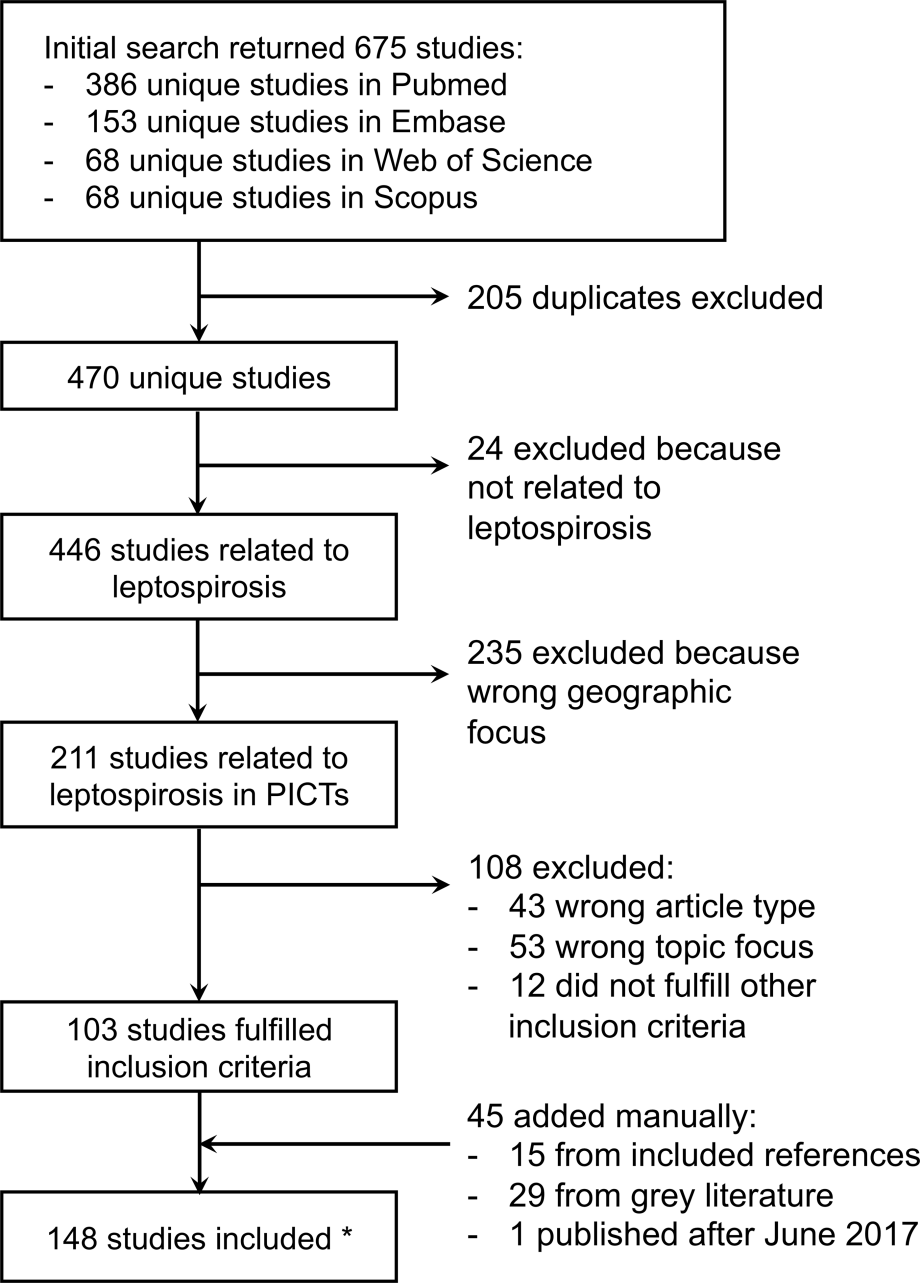
Flow diagram of the systematic review and identification of studies. *Among the 148 included studies, 11 full texts could not be retrieved but quantitative and/or qualitative data were collected from the abstract or from the full text of later published studies.

The initial search retrieved a total of 675 studies, including 386 on PubMed, 68 on Scopus, 68 on Web of Science, and 153 on Embase respectively. After removing 205 duplicates, 103 met the inclusion and exclusion criteria. A further 44 studies were identified, either from the reference list of already included papers (n = 15), or when searching internet websites for grey literature (n = 29). One publication from the last author of the present review (CL) that was accepted for publication after the search in June was also added, making a final list of 148 studies. For 11 studies, the full text document could not be retrieved but quantitative and/or qualitative data were available from the abstract, or from the full text of later published papers. The flow diagram of the search strategy is summarised in Fig 2.

The included leptospirosis studies were conducted in 21 out of the 25 PIs within the scope of our study. No information about leptospirosis was retrieved from Nauru, Pitcairn Islands, Tuvalu and Wake Island. Included studies were mostly either dedicated to animals (n = 54, 36.5%) or humans (n = 79, 53.4%), while very few studies (n = 13, 8.7%) provided information on both animals and humans. The remaining two studies (1.4%) focused on *Leptospira* in the environment [29, 30]. Detailed information on each study is provided in S1 Table.

### Human leptospirosis studies

**Fig 3:**
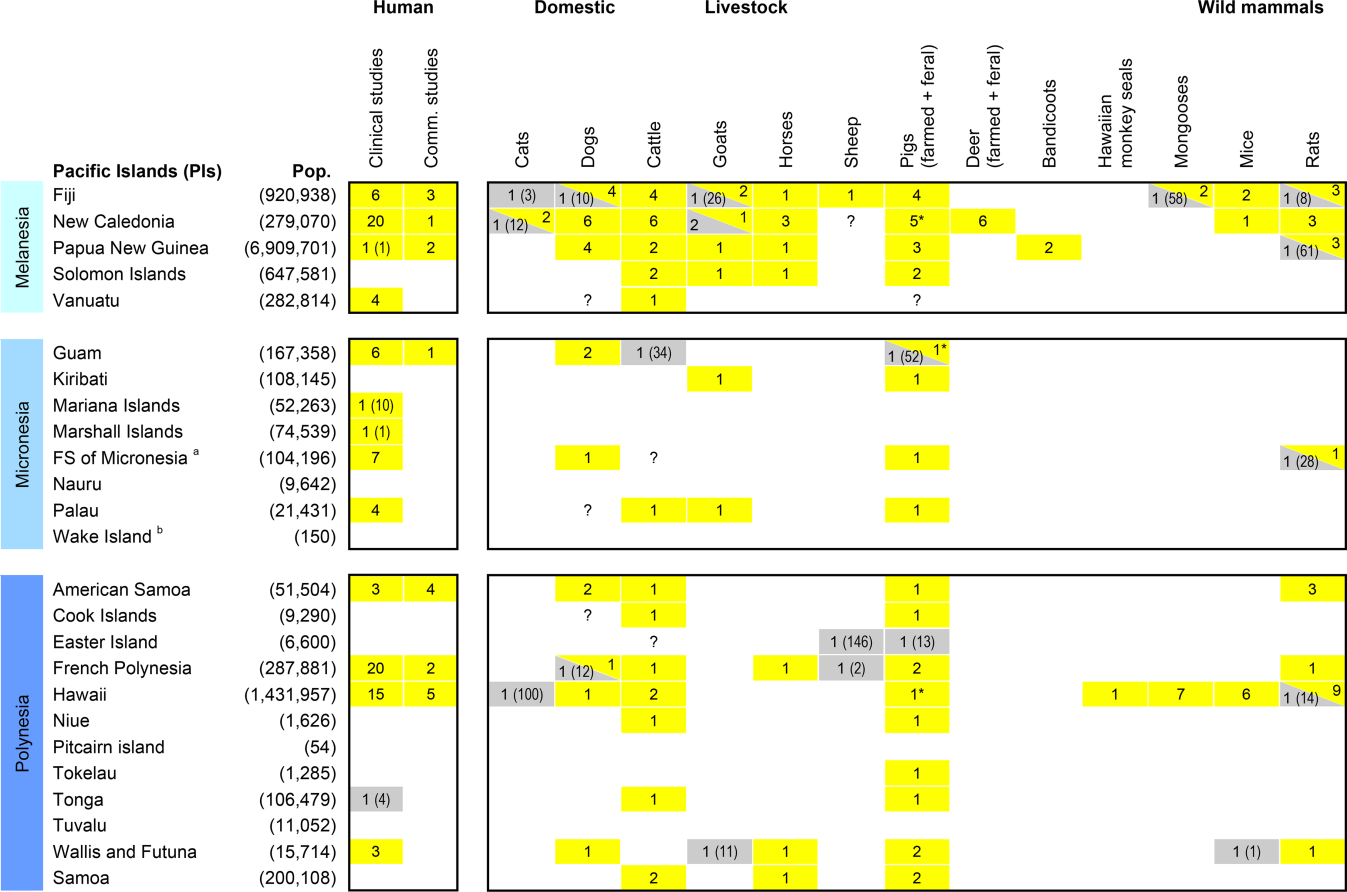
Number of studies that reported *Leptospira* infection from humans and/or animals in the Pacific Islands (PIs). Empty cells correspond to PIs/species for which no studies were retrieved; coloured cells and numbers correspond to PIs/species for which studies were retrieved, and the number of studies included in this review. The presence or absence of *Leptospira* is reported in yellow or grey respectively. For animal studies that provided no evidence of *Leptospira* infection, the number of tested animals is given in brackets to provide an indication of the degree of confidence of the negative results. Similarly, when only one human clinical study was conducted in a specific PI, the number of reported cases is given in brackets. ^a^ Federated States of Micronesia; ^b^ Wake Island is part of the United States Minor Outlying Islands. ‘Pop’: July 2016 estimated population size reported from the CIA World Factbook (https://www.cia.gov/library/) except for Hawaii, Wake Island and Easter Island for which data were retrieved from different internet sources. ‘Comm. studies’: human community-based studies. ‘*’: includes one study on feral pigs. ‘?’: inconclusive results (reported from [85]).

Human leptospirosis was investigated in 92 eligible studies from 14 PIs. We classified human studies into two categories: community-based studies (investigating healthy people) and clinical studies (investigating people who were unwell) (see Fig 3 and S1 Table). Community-based studies consisted of 19 (21%) seroprevalence studies, of which six were “mixed” studies that also included clinical case reports. The remaining 73 studies (79%) were clinical, including surveillance data, case reports or leptospirosis investigation in sick and/or hospitalised patients). Clinical studies were reported as strong or weak evidence of leptospirosis following the criteria detailed in the methods section (see S1 Table for detailed classification). French Polynesia was the most frequently represented PI with a total of 22 studies [5, 24, 31–48], followed by 20 from New Caledonia [32, 36, 42, 49–65] and 19 from Hawaii [66–84]. Of the 14 PIs investigated, confirmed cases were reported from 13 PIs only as four suspected leptospirosis cases investigated in Tonga were later reported as negative [5].

### Animal leptospirosis studies

Past infection with, or renal carriage of, leptospires in animals was investigated in 66 eligible studies from 19 PIs, plus one paper that reported animal leptospirosis from 22 PIs [85] (S1 Table). In total, 13 different animal species were tested: bandicoots (*Echymipera kalubu*), cats (*Felis catus*), cattle (*Bos* spp.), deer (*Rusa timorensis*), dogs (*Canis lupus*), pigs (*Sus scrofa*), goats (*Capra aegagrus*), horses (*Equus ferus*), mongooses (*Herpestes auropunctatus*), mice (*Mus musculus*), rats (*Rattus* spp.), sheep (*Ovis aries*), and in one case, Hawaiian monkey seals (*Monachus schauinslandi*). Both feral and farmed populations of pigs and deer were investigated. Livestock represented the majority of the investigations, i.e. farmed pigs (29 studies from 18 PIs) and cattle (27 studies from 14 PIs), followed by rats (28 studies from 8 PIs) and domestic dogs (24 studies from 9 PIs).

The majority of animal studies (n = 54, 82%) were seroprevalence studies. Renal carriage was also investigated (n = 14, 21%) especially in rats, either alone or in combination with serology. Older studies reported the presence of leptospires in kidneys from microscopic examination of tissues or by experimental infection of guinea pigs (e.g. by injection of crushed rat kidneys), while more recent studies used real-time PCR for confirmation of infection. Evidence of past or present *Leptospira* infection was most commonly found in farmed pigs (27 studies from 15 PIs) followed by cattle (24 studies from 13 PIs), rats (24 studies from 8 PIs) and dogs (22 studies from 9 PIs) (Fig 3). There was variation in the sample sizes between studies, ranging from one animal tested in some studies that reported on opportunistic sampling of animals to about 9,000 animals in a study of cattle in the Solomon Islands [86].

### Serology results by serogroup

Studies reported a total of 21 putative *Leptospira* serogroups in humans or animals across the Pacific, the most common being Icterohaemorrhagiae (15 PIs), Pomona (15 PIs), Australis (14 PIs) and Sejroe (13 PIs). Table 1 summarises the serogroups reported for humans and animal species for each PI. However, this finding should be interpreted with caution because of the many limitations of the MAT, including cross-reactions between serovars and serogroups, paradoxical reactions or anamnestic responses [2, 87]. MAT results might also vary depending on the panel of serovars used.

**Table 1:** Summary of serology results. Results are reported at the serogroup level. Human studies include both clinical and seroprevalence studies, while animal studies are seroprevalence only. A specific serogroup is reported as present in a PI if it was reported from at least one study over the 1947–2017 period, meaning that studies classified as ‘weak evidence’ for leptospirosis were also included. For this reason, and because of the limitations of the MAT, results should be interpreted with caution.

	Pacific Islands (PIs)	Serogroups	Australis	Autumnalis	Ballum	Bataviae	Canicola	Celledoni	Cynopteri	Djasiman	Grippotyphosa	Hebdomadis	Icterohaemorrhagiae	Javanica	Mini	Panama	Pomona	Pyrogenes	Santarosai	Sarmin	Sejroe	Shermani	Tarassovi
**Melanesia**	Fiji	human	✔	✔	✔		✔		✔				✔				✔				✔		
		animal	mice, cattle, goat, rat, mongoose, pig, sheep, cattle, dog, rat, horse	mice, pig, sheep, cattle, goat, rat, mongoose, horse, dog	cattle, pig, horse, dog, mice, mongoose	cattle, goat, rat, horse	cattle, horse, dog, pig	pig, horse	cattle, pig, horse	cattle, pig, horse		cattle, mongoose	cattle, goat, pig, horse, dog, rat	pig	cattle, mongoose	cattle	pig, goat, cattle, horse	cattle, goat, pig, horse, dog	cattle	pig, horse	cattle, goat, pig, horse, mongoose		cattle, horse, dog
	New Caledonia	human	✔	✔	✔	✔	✔		✔	✔	✔	✔	✔	✔	✔	✔	✔	✔			✔		✔
		animal	cattle, deer, horse, dog	cattle, deer, horse, dog	cattle, deer, horse, dog, cat	cattle, deer, cat	cattle, deer, horse, dog, cat		cattle, deer, horse, dog, cat				rat, cattle, pig, deer, horse, dog, cat			cattle, horse, cat	pig, deer, horse	cattle, horse, dog			pig, cattle, deer, dog		cattle, deer
	Papua New Guinea	human	✔	✔		✔				✔	✔	✔	✔				✔	✔					✔
		animal	dog, pig, goat, rat, cattle, bandicoot	dog, pig, goat, rat	goat, cattle, horse		rat, dog, pig, goat, horse		dog, pig	dog, pig	dog, pig, horse, rat	dog, pig, rat, cattle	dog, pig, rat				dog, pig, rat, cattle	dog, pig, goat, rat, cattle		dog, pig, bandicoot	dog, pig, cattle, horse, rat		dog, pig, cattle, horse, rat, bandicoot
	Solomon Is.	animal	cattle, pig, horse	cattle											cattle, horse		cattle, pig	pig			cattle, goat, horse		cattle, horse
	Vanuatu	human					✔			✔	✔		✔			✔							
		animal	cattle				cattle						cattle								cattle		
**Micronesia**	Guam	animal	feral pig										feral pig, dog				feral pig				feral pig		
	Kiribati	animal	pig?										goat, pig?				goat				goat		
	FSM	human	✔	✔	✔								✔										
		animal	dog, rat, pig	rat, pig	pig		pig		pig	pig	pig		dog, pig		pig	pig	pig	pig			dog		
	Palau	human											✔	✔									
		animal		goat			pig		pig		pig		pig		pig	pig	pig	goat, pig			cattle, pig	pig	
**Polynesia**	American Samoa	human	✔				✔					✔	✔				✔	✔					
		animal	rat?				dog						dog										
	Cook Is.	animal											pig				cattle						cattle
	French Polynesia	human	✔	✔	✔	✔	✔		✔	✔		✔	✔	✔	✔	✔	✔	✔	✔		✔		
		animal	cattle, pig, horse	cattle, pig		cattle, pig	pig		pig		cattle		cattle, pig, horse				cattle, pig, horse				cattle		cattle, pig
	Hawaii	human	✔	✔	✔	✔	✔					✔	✔	✔	✔		✔	✔			✔		
		animal	mongoose, feral pig, rat, cattle, HMS	feral pig	rat, mice, mongoose	cattle	mongoose	feral pig		feral pig			rat, feral pig, mongoose, mice, HMS		feral pig		feral pig, HMS	feral pig			cattle, HMS, rat, mice, mongoose		
	Tokelau	animal							pig									pig					
	Tonga	animal	pig	cattle								cattle	pig				cattle, pig				cattle		cattle
	Wallis and Futuna	human	✔	✔	✔		✔																
		animal	dog, pig	dog, pig					dog, pig	dog	dog, pig		dog, pig			pig	dog, pig	pig				pig	pig
	Samoa	animal	cattle, horse, pig	cattle, pig	cattle, pig	horse, pig	cattle, horse, pig		horse, pig	pig		cattle, horse	cattle, horse, pig	cattle, horse	cattle, horse, pig	cattle, horse	cattle, pig	cattle, horse, pig			cattle, pig	cattle	cattle, pig

### Hawaii

Human leptospirosis was recognised as early as 1936 in Hawaii; seroprevalence studies investigated the general population in Honolulu [66] and sugar cane workers [67] in 1936–1942, and revealed seroprevalence rates of 3.8% (13/344) and 12.2% (105/860) respectively, using a cut-off titre of 1:100. A seroprevalence study of US army blood bank donors conducted in 2002 in Oahu found a positivity rate of 1.4% (7/488) [79]. Sixteen publications reported leptospirosis cases from surveillance data and clinical studies between 1962 and 2008. Information by island was not always available as surveillance data generally included cases from all islands, but a few studies were dedicated to Kauai and Oahu, reporting sporadic cases. One study investigated cases of leptospirosis during an outbreak of murine typhus on Kauai (1998) and reported two cases of co-infection with both diseases [76]. The complete list of human studies from Hawaii is reported in Table 2.

**Table 2:** Summary of publications reporting human leptospirosis in Hawaii, 1947–2017.

Citation	Study years	Case reports	Seroprevalence studies	Exposure/risk factors
[66]	1936–42	Hawaii: 59 cases; Kanai: 4 cases; Lanai: 14 cases; Maui: 1; Oahu: 4	Honolulu residents: 13/344 (3.8%)	N/A
[67]	1943?		Workers from sugar cane plantations: 105/860 (13.0%)	Outdoor work, field work; heavy rainfall
[68]	1962–63	3 cases		Exposure: Waded barefoot in a creek
[69]	1960–75	Hawaii: 100 cases; Oahu: 21 cases; Kauai: 7 cases; 19 cases hospitalized		N/A
[70]	1970–84	186 confirmed cases		Prior to 1950: occupational risk (sugar industry); after 1950: recreational risk
[71]	1987	Kauai: 16 cases		Swimming in the Waimea river
[72]	1988–89	Hawaii: 20 confirmed / 172 suspects Kauai: 13 confirmed / 100 suspects		Use of water catchment systems, skin wounds, handling animal tissues (especially cattle)
[73]	1992	Oahu: 2 confirmed cases		Recreational exposure (falls); not linked to rats
[74]	1989–97	7 cases		N/A
[75] [77]	1974–98	353 confirmed / 752 suspects		Decrease of occupational exposures over time; increase of recreational exposures. Animal exposure: dogs 71%, rats 30%. Largest outbreak: swimming in a river
[76]	1998	Kauai: 2 cases / 5 murine typhus cases		N/A
[78]	1979–98	74 confirmed cases		N/A
[79]	2002		US Army blood donors, Oahu: 7/488 (1.4%)	N/A
[80]	2004	Oahu: 2 confirmed / 48 tested		Flooding event on a university campus
[81]	2001–2	54 confirmed /1206 febrile illness *		N/A
[83]	2011?	1 case		Cliff-diving in Maunawili Falls
[82]	1992–2004	18 confirmed cases		Freshwater exposure (83%); 50% of cases acquired infection in Hawaii, others were infected in Japan, Malaysia and other PIs.
[84]	1999–2008	198 confirmed / 345 suspects		Recreational exposure 45%; Occupational exposures 44%; Habitational exposure 11%

N/A: no information available from the study.

*The study was conducted during a dengue outbreak.

In Hawaii, the epidemiology and relative importance of risk factors for human leptospirosis have evolved over the past few decades. Leptospirosis in Hawaii has historically been considered an occupationally-acquired disease affecting primarily sugarcane labourers and farmers, but a shifting trend in exposure has been observed since the 1970s, with increasing importance of recreational exposure (freshwater swimming, hunting, fishing, hiking) [70, 75, 77]. During 1989–2008, the frequency of recreational exposures plateaued while frequency of occupational exposures seemed to increase (at least for the island of Hawaii). At the same time, a significant shift in the seasonal occurrence of leptospirosis from the drier summer months to the wetter winter months was observed [84]. An increase in “habitational risks”, especially gardening at home, was suspected to be linked to the resurgence of traditional taro farming. Lastly, exposure to feral pigs, although not investigated *per se* in the epidemiological surveys, was suspected to be responsible for the changing trend in the infecting serogroup in human cases, shifting from Icterohaemorrhagiae to Australis.

Animals were first investigated for leptospirosis in Hawaii during the same 1936–1942 investigation targeting humans. Dogs, cats, rats and mongooses were serologically screened for leptospirosis, and revealed evidence of infection in all three species [66, 88]. Between 1943 and 2009, more animal studies followed, investigating six animal species. Seropositive rats and mongooses were reported from nine and seven more studies respectively. Evidence of leptospirosis infection was also found in cattle (2/2 studies), mice (6/6), feral swine (1/1) and Hawaiian monkey seals (1/1). Surprisingly, dogs and cats were never further investigated. The complete list of animal studies from Hawaii is reported in Table 3.

**Table 3:** Summary of publications reporting animal leptospirosis in Hawaii, 1947–2017.

Citation	Study years	Animal species	Test	N	Positive cases (%)
[88]	N/A	Dogs	S	100	39 (39%)
[66]	1936–42	Cats	S	100	0
		Rats	DF	447	18 (4%)
		Mongooses	DF	12	4 (33%)
[67]	1943	Rats	S, DF	96	17.2% (Kohala district), 31.3% (Olaa district)
		Mongooses	S, DF	86	20% (Kohala district), 23% (Olaa district)
[89]	1950s-60s	Rats, mice, mongooses	S	1238	558 (45%)
[90]	1959–61	Rats (*RR*, *RN*, *RE*)	S	1705	587 (34%)
		Mice	S	170	98 (58%)
		Mongooses	S	152	49 (32%)
[91]	1970–73	Rats (*RR*, *RN*, *RE*)	S, DF, IC	819	201 (25%)
		Mice	S, DF, IC	26	5 (19%)
		Mongooses	S, DF, IC	282	65 (23%)
[92]	1969–74	Rats (*RR*, *RN*, *RE*)	S, IC	305	50% *RN*, 55% *RR*, 34% *RE*
		Mice	S, IC	53	33%
		Mongooses	S, IC	180	23%
[93]	1969–73	Rats (*RR*, *RN*, *RE*)	S	2389	722 (30%)
		Mice	S	95	41 (43%)
		Mongooses	S	473	136 (29%)
[71]	1987	Rats	DF	14	0
		Cattle	S	139	89 (64%)
[94]	N/A	Cattle	S, IC	-	1 isolation from kidney
[73]	1992	Rats (*RR*, *RN*, *RE*)	S, IC	85	4/77 *RR*, 1/2 *RN*, 0/6 *RE*
[76]	1992–98	Mongooses, rats, mice	S, IC	12501	2391 (19%)
[95]	1997–2001	Hawaiian monkey seals	S	308	19 (6%)
[96]	1990–2003	Rats, mice	S, DF, IC	15171	2766 (18.2%)
[97]	2007–2009	Feral swine	S	804	33.8%

N/A: information unavailable from the study; N: number of animals tested; *RR*: *Rattus rattus*; *RN*: *Rattus norvegicus*; *RE*: *Rattus exulans*; S: serology test; DF: microscopic examination of kidney tissues with a dark field microscope; IC: isolation by culture.

Hawaii is one of the two places (with New Caledonia) where leptospires were investigated in the environment. A molecular study from 2011 focused on environmental samples collected in 22 tropical streams of Oahu near the point at which they discharge to the coastal ocean, showing evidence of *Leptospira* in 87 of 88 samples. All of the sequenced amplicons (n = 42) were characterised as pathogenic *Leptospira wolffii*.

### New Caledonia

Human leptospirosis was first reported in 1954 and 1957. A total of 20 articles from New Caledonia were identified. Two seroprevalence studies were conducted in 1985–86 [53, 54], and the majority of articles were clinical studies (n = 18) including case reports, routine surveillance data, and case investigations. Clinical studies were conducted from 1973 to 2012, with frequent reports except between 1990–1998, suggesting endemic transmission. Three clinical studies focused on severe leptospirosis: severe icteric leptospirosis cases with cardiac manifestations [55], the influence of age on the development of severe leptospirosis in children [63], and risk factors and predictors of severe leptospirosis [64]. The complete list of human leptospirosis studies from New Caledonia is reported in Table 4.

**Table 4:** Summary of publications reporting human leptospirosis in New Caledonia, 1947–2017.

Citation	Study years	Case reports	Seroprevalence studies	Exposure/risk factors
[32]	1954, 1957	7 confirmed cases		N/A
[36]	1974–79	24 confirmed cases		N/A
[49]	1973–80	32 cases		N/A
[50]	1973–80	37 confirmed / 286 suspects		Possible drivers: Poor hygiene, rivers
[51]	1973–81	39 confirmed cases 1981: 5 cases (1 infected in Vanuatu)		Possible exposure: Streams, occupational activities
[52]	1983–85	57 cases		Living in rural areas, in tribes
[53]	1985–86	193 cases	Around cases: 63/210	Cases: 72% live in flood plains
			Occupational risk: 124/669	Around cases: farmers most at risk
			Control population: 26/260	Occupational risk: farmers most at risk
[54]	1985–86	La Néra: 60 cases	Around cases: Néra = 16/41; Coulée = 5/20	Cases: 85% live in a muddy place; 95% in frequent contact with rivers
		La Coulée: 26 cases	Occupational risk: Néra = 16/41; Coulée = 16/93	
[55]	1983–85	57 cases, 15 with cardiac symptoms		N/A
[42]	1987	46 cases		N/A
[56]	1989–93	192 confirmed cases Peak in 1990 (51 cases)		Temporality: 4-fold increase in March 65.9% cases: Melanesian leading a tribal lifestyle; subsistence farming Recreational risks: bathing in running fresh water, fishing, hunting
[57]	1989	144 confirmed cases		Exposure: ditch or river water; living in a rural area. Contacts with rats, dogs
[58]	1989–90	Bourail: 78 confirmed cases		Exposure: Swimming, poor hygiene, hunting, fishing, contact with animals
[60]	1989–2001	156 cases		
[59]	2005	37 confirmed and 3 probable / 1059 suspects		Exposure: Swimming, hunting, fishing, contact with animals
[61]	2001–05	239 confirmed / 6690 suspects		Contacts with animals, recreational activities
[62]	2008	Outbreak: 135 confirmed cases		Suspected driver: heavy rainfalls associated with floods. Exposure: Contact with animals (OR>2), swimming, fishing, hunting (OR>3)
[63]	2006–12	60 cases under 18yo		Age-dependant association with severity of leptospirosis
[64]	2008–11	72 severe leptospirosis / 306 cases		Delay in diagnosis, tabacco use, infection by *L*. *interrogans* Icterohaemorragiae
[65]	2000–12	731 confirmed and 432 probable/ 1163 suspects		SST anomaly, rainfall, temperature

N/A: information unavailable from the study.

The risk factors for human leptospirosis in New Caledonia varied little between the 1980s and today. The disease was more frequent among young men and during the wet season. The main risk factors identified were recreational exposure (fishing and swimming in fresh water, hunting) and contact with animals, with populations living in rural areas and local tribes being at highest risk. However, the probable source of infection was difficult to ascertain because of the multiplicity of potential infecting sources and exposure pathways, and the overlap between professional and recreational activities [56]. Also, identification of the animal source of leptospires in these (human only) studies was only presumptive as people were frequently exposed to multiple species, including cattle, pigs, horses, dogs, rats and, less commonly, deer [56, 61].

Eleven studies were published on animal leptospirosis in New Caledonia between 1983 and 2004. *Leptospira* spp. infection was demonstrated in a wide range of animal hosts, including cattle (6/6 studies), dogs (6/6), deer (6/6), horses (3/3), pigs (4/5, including one study of feral pigs), rats (3/3) and cats (2/3). Results on goats largely lacked detail, however one study reported five seropositive animals. Results on sheep (one study) were inconclusive. The complete list of studies on animal leptospirosis in New Caledonia is reported in Table 5.

**Table 5:** Summary of publications reporting animal leptospirosis in New Caledonia, 1947–2017. For study [54] the number of tested animals ‘N’ is provided separately for the two areas of interest, i.e. La Néra basin first and La Coulée basin then. Studies [60] and [98] targeted positive animals only, so ‘N’ equals ‘positive cases’.

Citation	Study years	Animal species	Test	N	Positive cases (%)
[99]	1982–83	Cattle	S	136	84 (61.8%) seropositive
		Dogs	S	38	12 (31.6%) seropositive
		Horses	S	25	13 (52%) seropositive
		Pigs	S	43	10 (23.3%) seropositive
		Sheep/goats	S	2	1 (suspected) seropositive
[100]	1986	Cattle	S	5	5 seropositive
[53] Grande Terre and Islands	1985–86	Rats (*RR*, *RN*, *RE*)	S, DF, IC	18	11 (61.1%) seropositive
		Cattle	S, DF, IC	70	69 (98.7%) seropositive
		Pigs	S, DF, IC	36	21 (58.3%) seropositive
		Horses	S, DF, IC	18	9 (50%) seropositive
		Goats	S, DF, IC	13	N/A
		Dogs	S, DF, IC	81	48 (59.3%) seropositive
		Cats	S, DF, IC	12	NA
[54] La Néra + La Coulée	1985–86	Rats (*RR*, *RN*, *RE*)	S	12 + 4	8 + 3 seropositive
		Cattle	S	15 + 0	15 seropositive
		Pigs	S	14 + 0	?
		Horses	S	4 + 0	?
		Goats	S	9 + 4	1 + 2 seropositive
		Dogs	S	46 + 7	29 + 2 seropositive
		Cats	S	6 + 5	0 + 1 seropositive
		Deer	S	1 + 0	1 seropositive
[101]	1984	Cattle	S	350	58.3% seropositive
[60]	1989–2001	Dogs	TY	1	1
		Pigs	TY	7	7
		Deer	TY	5	5
[98]	2010	Introduced deer	TY	12	12
[102] Poster summary		Introduced deer			yes
		Rats (same as [103])			yes
[103]	2008–10	Rats, mice (Bourail area)	MT	210	56 (26.7%) renal carriage
[104]	2009	Cattle	S	30	43% seropositive
		Deer	S	29	72% seropositive
		Horses	S	25	80% seropositive
		Dogs	S	51	43% seropositive
		Cats	S	8	100% seropositive
[105]	2013	Dogs (urban + rural)	S, MT	95	30.8% seropositive; 4.4% renal carriage
		Pigs (feral + farmed)	MT, TY	94 + 138	6.4% (feral); 10.2% (farmed)
		Deer (feral + farmed)	MT, TY	85 + 107	13.02%

N/A: information unavailable from the study; N: number of animals tested; *RR*: *Rattus rattus*; *RN*: *Rattus norvegicus*; *RE*: *Rattus exulans*; S: serology test; DF: microscopic examination of tissues with a dark field microscope; IC: isolation by culture; MT: molecular testing (PCR); TY: molecular typing.

In one study from 2016 in New Caledonia, leptospires were investigated in the environment [29]. The study focused on environmental samples collected around human cases, showing that 58% of soil samples were contaminated with pathogenic leptospires.

### French Polynesia

The first human leptospirosis cases were reported from Tahiti in the 1950s [31]. Two serological surveys in the general population were conducted in French Polynesia: one in Tahiti in 1970–71 [34] found a seroprevalence of 29.5%, and another in Marquesas in 1981 [39] where a lower seroprevalence (9.5%) was found. In addition, 20 clinical studies from 1952 to 2015 provided evidence of human leptospirosis, suggesting endemic transmission throughout the archipelago. Available reports generally covered the whole of French Polynesia, a vast archipelago of 118 islands and atolls, because diagnostic laboratories received samples from clinically-suspected cases from all islands, but a few studies were dedicated to specific Islands, i.e. Marquesas [5, 39, 46] and Raiatea [5, 46]. The complete list of human studies from French Polynesia is reported in Table 6.

**Table 6:** Summary of publications reporting human leptospirosis in French Polynesia, 1947–2017.

Citation	Study years	Case reports	Seroprevalence studies	Exposure/risk factors
[31]	1952–53	Tahiti: 2 cases		Rats trapped in the same suburbs are infected; pigs and dogs might be
[32]	1952–57	7 confirmed + 4 probable / 35 suspects		N/A
[33]	1969–70	Tahiti: 36 cases		N/A
[34]	1970–71		Tahiti: 224/759 (29.5%)	N/A
[35]	1955–68 1969–72	22 confirmed cases 66 confirmed cases		Possible drivers: favorable climatic conditions; flood-risk areas; lots of rats and roaming dogs
[36]	1970–79	77 severe cases		N/A
[37]	1975–80	43 confirmed / 60 hospitalized		Occupational risk (16), rodents around (most); 50% of cases don't swim in rivers
[38]	1975–80	60 cases		Thrombocytopenia (16), renal failure (15), both (10)
[39]	1981		Marquesas: 24/253 (9.5%)	Exposure: contacts with dogs, rats; walking bare-foot
[40]	1983	11 cases		N/A
[41]	1985–87	Tahiti: 6 cases with pulmonary complications		N/A
[42]	1987	36 cases		N/A
[43]	1984–90	114 confirmed / 120 hospitalized		Exposure: living in rural or semi-rural areas in contact with livestock (pigs)
[44]	1994–2000	360 confirmed cases / 2525 suspects		N/A
[45]	2004–05?	Tahiti: 71 severe cases		Severe outcome linked to hypotension, oliguria, abnormal chest auscultation at the first examination
[5]	2003–05	Marquesas: 13 confirmed /47 suspects		Marquesas: hunting (75% cases)
		Raiatea: 16 confirmed /68 suspects		Raiatea: bathing if freshwater (64% cases)
[46]	2004–05	Marquesas: 13 confirmed /47 suspects		Possible drivers: Contact with dogs, hunting in Marquesas; contacts with rats in Raiatea
		Raiatea: 20 confirmed /66 suspects		
[47]	2006–08	165 confirmed cases + 107 probable		Swimming in river (30%), farmers (22%), contact with rats (24%) dogs (21%) or cats (17%), walking barefoot (21%), gardening (18%)
[106]	2010	81 confirmed cases		Contact with rats (40%) or domestic animal (60%), walking barefoot in water or mud (77%); swimming in rivers (43%), gardening (50%); working in piggeries (17.5%), farmer (52%)
[107]	2006–10	502 cases, 100 serotyped		Contact with rats (34%) or domestic animal (60%), walking barefoot in water or mud (33%); swimming in rivers (35%), gardening (30%); working in piggeries (14%), farmer (29%)
[24]	2014–15	2 cases (co-infection with chikungunya)		N/A
[48]	2014–15	44 confirmed cases		Possible risk factors: rats and dogs (as they share common types of leptospires with human cases)

N/A: information unavailable from the study.

Three clinical studies included case investigations (through questionnaires) that aimed to identify risk factors associated with human leptospirosis (2006–2010), and important factors common between the studies included contact with rats or domestic animals, walking barefoot in water or mud, swimming in rivers, gardening, and occupational risks (working in piggeries, farming) [47, 106, 107]. In Marquesas, hunting and contact with dogs have been reported as possible sources of infection [5, 46]. Risk factors in French Polynesia were mostly recreational rather than occupational, but potential infecting sources were numerous.

Only three studies investigated animal leptospirosis in French Polynesia. The first study (1952–53) investigated renal carriage in rats from Tahiti by experimental infection of guinea pigs with crushed rat kidneys; four guinea pigs became icteric [31]. The second was a seroprevalence study (1981–86) targeting four host species from Tahiti, and cattle from both Tahiti and Marquesas [108]. Positive serology results were found in 15.5% (23/148) of cattle, 32.2% (37/115) of pigs, and all five horses tested. Serology results were not detailed by island, but only seven tested cattle originated from Marquesas. The two sheep and twelve dogs tested were found to be seronegative. The last study investigated animal leptospirosis in dogs, pigs and rats from Tahiti [48]. Renal carriage of pathogenic leptospires was demonstrated from 26.5% (48/181) of farmed pigs, 20.4% (23/113) of rats, and four sick dogs.

### Fiji

The first study reporting human leptospirosis in Fiji was published in 1978 [109], describing 240 cases between 1969 and 1977. Since then, two community-based seroprevalence studies were published [110, 111], as well as five studies reporting clinical cases [5, 112–115]. The first seroprevalence study in the 1980s identified 264 seropositive out of 300 healthy volunteers (88%) from a rural area in the main island of Viti Levu [110]. A second seroprevalence study was conducted in 2013 and included 2,152 healthy individuals from 81 communities on the three main islands of Fiji, of whom 19.4% were seropositive [111]. Risk factors associated with infection included living in villages (OR 1.64), lack of treated water at home (OR 1.52), working outdoors (1.64), living in rural areas (OR 1.43), high poverty rate (OR 1.74), living <100m from a major river (OR 1.41), pigs in the community (OR 1.54), high cattle density in the district (OR 1.04 per head/km^2^) and high maximum rainfall in the wettest month. Using the data from the 2013 seroprevalence study (which included questionnaire data about contact with animals as well as livestock data from the Fiji Ministry of Agriculture), a third study [116] showed significant heterogeneity in the relative importance of animal species in leptospirosis transmission in different ethnic groups and residential settings.

Five studies have been published on animal leptospirosis in Fiji [113, 117–120], exploring a wide range of host species, including dogs, goats, mongooses, cattle, pigs, rats, sheep, mice, horses and cats. Except for cats (only three animals tested), the studies identified seropositive animals in all species that were tested. Thus, *Leptospira* infection seems common in multiple animal species in Fiji.

### Papua New Guinea

Studies on human leptospirosis in Papua New Guinea (PNG) were published between 1955 and 1968 [121–124], including three seroprevalence studies targeting healthy volunteers, showing high seroprevalence of 53.0% to 57.5%, with Australis and Hebdomadis the most common serogroups. One study explored risk factors for human infections and found that leptospirosis in PNG was common in both males and females, and in both adults and children, reflecting high risk in the whole population from shared occupational, domestic, and other environmental exposures [123]. People have close contact with domestic animals, particularly dogs and pigs, as well as native fauna in gardens and uncleared areas surrounding the villages. Rats were suspected to be of less importance compared to other animal species because human studies in PNG found that serogroup Icterohaemorrhagiae (commonly associated with rodents) was relatively uncommon [122, 125].

*Leptospira* infection in animals was explored in the 1960s and 1970s, with seven publications exploring infection in rats, cattle, dogs, pigs, goats and marsupial bandicoots [122, 123, 125–129]. Seropositive animals were identified from all tested species, but sample size was small for bandicoots (n = 5). After the 1970s, no further studies were published until a seroprevalence study investigated cattle (n = 1,452) and pigs (n = 326) in 2004, and dogs and livestock in 2006 (111 cattle, 69 pigs, 22 dogs, 15 horses) [130]. Seropositive animals were identified from all species, but seroprevalence was low in dogs (1/22 in 2006) and pigs (0/362 in 2004, 1/69 in 2006).

### Guam

The first study published in 1974 identified three seropositive stray dogs out of 180 tested (1.7% seroprevalence) [131]. More recent studies investigated cattle (n = 34) and pigs (n = 52) in 1999 [132], and feral pigs (n = 46) in 2015 [133]. All livestock tested seronegative but the seroprevalence in feral pigs was 23.9%.

The first human clinical study was published in 1998, describing two human leptospirosis cases with pancreatitis [74]. Three human clinical studies in the 2000s reported sporadic cases only [5, 74, 134, 135]. In addition, two leptospirosis cases reported in Hawaii for the period 1999–2008 were suspected to have been acquired in Guam [84]. One mixed study included both clinical data and a seroprevalence study conducted following an outbreak that occurred after an outdoor multisport athletic event: out of 46 participants surveyed, 21 reported being ill and three of them were confirmed with recent leptospirosis infections [136]. Reported exposures were often recreational activities involving swimming in fresh water or waterfalls, but water buffaloes were also reported as a suspected source of contamination.

### Wallis and Futuna

No seroprevalence study has been conducted in Wallis and Futuna. A multicentre survey of suspect clinical cases recruited by general practitioners conducted in 2003–2005 identified 3 cases out of 14 (21%) and 31 cases out of 71 (44%) in Wallis Island and Futuna Island respectively [5]. Another clinical study conducted in 2005 identified one case in Wallis and 21 cases in Futuna [137], and reported some exposure information: one case swam in a river few days before the occurrence of the disease, and all the other cases were involved in breeding pigs (at home for the Futuna cases, at work in a pig farm for the one Wallis case). A third study conducted over the period 2004–2014 reported 382 cases in Futuna, with a peak incidence in 2008 [7]. Serogroup Australis was predominant until 2007, when Icterohaemorrhagiae became the most common. Despite similar cultural and socio-economical patterns between the islands, human leptospirosis was considered endemic in Futuna while it only occurred sporadically in Wallis.

Three studies focused on animal leptospirosis in Wallis and Futuna. A first study focusing on livestock only reported the detection of specific *Leptospira* serogroups in pigs, with no prevalence provided [138]. A seroprevalence study was conducted in the 1990s on different animal species: 14% (12/88) of pigs were found to be seropositive in 1985, and 33% (54/163) in 1997 but a wider range of serogroups was tested in 1997. In 1998, two dogs out of 10 and three horses out of six also tested seropositive, while all 11 goats tested were seronegative [138, 139]. In 2008–2012, *Leptospira* prevalence in three rat species was investigated [140]. Renal carriage of *Leptospira interrogans* was confirmed in 84 rats out of 286 in Futuna (36.4% *Rattus rattus*, 42.8% *Rattus norvegicus* and 18.8% *Rattus exulans*), while only one *R*. *exulans* out of 56 rats (1.8%) was positive in Wallis; 15 rats were found negative in the uninhabited island of Alofi, 2 km from Futuna.

Risk factors associated with human leptospirosis have not been formally investigated, but rodents in Futuna have a much higher *Leptospira* carriage rate than on Wallis. It has been hypothesised that this variation could be explained by differences in taro farming practices; people on Wallis irrigate their fields by ditches, while those on Futuna flood their fields. This practice in Futuna might facilitate the spread of *Leptospira* among rats and subsequently to humans [140].

### American Samoa

Human leptospirosis was first investigated in American Samoa in 1948 [141]; ten patients hospitalised for jaundice were tested, but none were seropositive against four *Leptospira* serogroups. Thirty patients hospitalised for reasons other than jaundice were also tested, and three were found to be seropositive for serogroup Australis. Leptospirosis cases were later occasionally reported, including three cases of co-infection with dengue in 2008 [142] and one severe case in 2011 of a 15 year old boy who frequently slept in the rainforest and had recently waded through freshwater [143].

Four community-based seroprevalence studies were conducted in American Samoa. Two studies reported seroprevalence rates of 17% in 2004 (n = 341) [144] and 15.5% in 2010 (n = 807) [145]. Australis was the main serogroup in 2004 (71%) while Hebdomadis, Australis and Pyrogenes were the most common serogroups in 2010. The 2010 survey found that outdoor occupation (OR = 3.25), piggeries around the house (OR = 2.63) and recreational exposure (swimming at beach OR = 2.01, fishing OR = 1.78) were significantly associated with seropositivity, but infections with each of the three main serogroups were associated with different behavioural and environmental exposures [145]. A third study compared the serogroups between the 2004 and 2010 surveys [146] and showed epidemiological evidence of serogroup emergence, possibly as a result of ecological and environmental change. The fourth study produced a predictive risk map using environmental variables from 2010 study and demonstrated the importance of environmental drivers of transmission [147].

Two studies focused on animal leptospirosis. Renal carriage of leptospires by rats was examined on Tutuila Island in 1948; out of 126 individuals from four rat species trapped, 24 (19%) *Rattus norvegicus* kidney tissues stained by silver precipitation technique were found positive under microscopic examination [141]. One out of twelve dogs was also found seropositive by MAT. A more recent study published in 2005 reported data ‘from animal studies and literature’ (with no date specified) showing seropositive results from pigs, rats, dogs and cattle [135]. The authors suggested that, even though the animal studies were not conducted at the same time as the human studies (2004), it appeared that Australis was the most common serogroup infecting people, and pigs were the most likely reservoir hosts.

### Federated States of Micronesia

Seven clinical studies reported information about human leptospirosis in the Federated States of Micronesia (FSM), which consists of the four states of Yap, Chuuk, Kosrae and Pohnpei in the Northwestern Pacific Ocean. In 1989–1997, eight confirmed leptospirosis cases identified in Hawaii were acquired from Kosrae and Pohnpei [74], and from 1999–2008 one reported case in Hawaii was acquired from FSM [84]. A multi-centre survey of patients with suspected leptospirosis conducted in 2003–2005 reported no seropositive case from Yap (0/1 suspected) or Pohnpei (0/27 suspected) [5]. In 2010, an investigation of 10 febrile patients in Chuuk revealed two confirmed cases of leptospirosis [148]. In 2011, a hospital-based survey conducted in Pohnpei on 54 patients presenting with undifferentiated fever found that 20.4% showed serologic evidence of acute infection by MAT [149]. In 2012 in Yap, 172 patients with suspected dengue were investigated by qPCR and five (2.9%) were confirmed as acute leptospirosis infections [150]. Lastly, one study investigated the health risks associated with climate change in FSM using a time series distribution of monthly leptospirosis outpatient cases in Pohnpei. This study was based on hospital records and is assumed to represent close to all of the reported cases [151]. Taken together, those studies suggest that leptospirosis is endemic in FSM.

Rats were the first animal species to be investigated in 1947, but microscopic examination of kidney tissues stained by silver precipitation technique did not reveal any leptospires from 28 rats trapped in Chuuk and Pohnpei [152]. However, a seroprevalence study conducted in 1995–1996 on pigs (in four states), dogs (Pohnpei, Chuuk) and rats of three species (Pohnpei, Chuuk, Yap) showed positive results from all species [153].

### Solomon Islands

No papers on human leptospirosis were identified from the Solomon Islands, and only three papers on animal leptospirosis have been published. Between 1967 and 1977, a large veterinary survey investigated cattle diseases from all the cattle herds listed in 1967 (165 herds with a total of 8,930 cattle) plus some of the 650 herds established after 1967 [86]. For leptospirosis, only the female cattle over one year of age were tested, of which 62 were found seropositive. In 1985, pigs were reported positive with serogroup Pomona, but detailed results were not available [154, 155]. A seroprevalence study conducted in 1998 reported that 83% of 226 cattle, 12% of 298 pigs, 16% of 63 goats, and 71% of 31 horses tested were seropositive by MAT [155].

### Vanuatu

Only sporadic cases of human leptospirosis have been reported in Vanuatu in the 1990s [156, 157] and the 2000s [5]. No human seroprevalence studies have been conducted. Two cases in travellers returning from Vanuatu were also reported in New Caledonia [51] and Australia [158]. The latter swam in a river in Vanuatu with an injured foot, and developed a fatal illness with acute renal failure, jaundice, respiratory failure, myocarditis and rhabdomyolysis.

During an extensive survey of livestock diseases conducted between 1971 and 1981 throughout the Vanuatu archipelago, 6,719 cattle from 131 herds were investigated by MAT; 92 cattle (1.4%) were found positive [159].

### Palau

Following the presentation of three cases of Weil’s disease in 2000, 171 patients presenting with a ‘viral syndrome’ were investigated in Palau, of whom seven were serologically confirmed as leptospirosis [160]. Over the period 2000–2006, the disease surveillance system recorded 81 cases, all living in the most populated areas of the country [161]. In 2003–2005, a multi-centre survey of patients with clinically suspected leptospirosis reported one seropositive out of eight tested [5]. In 2014, two Japanese travellers developed leptospirosis after returning from Palau; the suspected exposure was swimming in Ngardmau falls [15].

Cattle (n = 20), goats (n = 7) and pigs (n = 55) were serologically investigated by MAT between 1993 and 1996 in Palau [162]. All three investigated species were found seropositive for *Leptospira*, i.e. 9 cattle (45%), 3 goats (43%) and 22 pigs (40%).

### Other Pacific Islands

In Tonga, a multi-centre survey of clinically suspected cases was conducted in 2003–2005; four patients were tested by PCR or MAT but none were confirmed as leptospirosis [5]. Cattle (n = 171) and pigs (n = 244) from different Tongan islands (Tongatapu, 'Eua, Ha'apai, Vava'u) were serologically investigated by MAT between 1992 and 1994 [163]. Seroprevalence varied between the islands, from 19.6% to 45.0% in cattle, and from 5.0% to 16.7% in pigs.

In the Commonwealth of the Northern Mariana Islands (CNMI), leptospirosis has been reported as endemic, but our search retrieved only one study; in 2000–2001, 10 cases of leptospirosis were reported in Saipan, of which eight were severe, and three were fatal [164]. In none of the cases was laboratory diagnosis available to the medical staff until weeks after patient was hospitalized or had died. Case reports available for four of the patients identified possible exposures as swimming in freshwater, cleaning out roadside sewers after a tropical storm, slaughtering pigs, and occupational gardening.

In 1989–1997, one confirmed leptospirosis case identified in Hawaii was acquired from the Marshall Islands [74].

For the remaining Pacific Islands, our search retrieved no studies on human leptospirosis, but few animal studies were available for Easter Island, Kiribati, Niue, Cook Islands, Samoa and Tokelau. The results are summarised in Table 7.

**Table 7:** Summary of publications reporting animal leptospirosis in the Pacific Islands for which no studies on human leptospirosis were reported, 1947–2017.

Citation	PICTs	Study years	Host	N	Confirmed cases (%)
[165]	Easter Island	1946–65	Cattle	57	46 low-grade reactions with MAT
			Sheep	146	0
			Pigs	13	0
[166]	Kiribati	1975–80	Pigs	172	0
		1985	Goats	51	8 (15.7%) seropositive
		1992–94	Pigs	193	6 (3.1%) seropositive
[167]	Niue	1992–94	Cattle	60	Inconclusive serology
			Pigs	60	Inconclusive serology
[168]	Cook Islands (Raratonga)	1982–83	Cattle	48	3 (6.3%) seropositive
			Pigs	60	1 positive, 6 inconclusive (11.7%)
[141]	Samoa	1948?	Rats	36	8 (22.2%) renal carriage
[169]	Samoa	?	Cattle	629	414 (65.8%) seropositive
			Pigs	108	27 (25%) seropositive
[170]	Samoa	1997	Cattle	316	125 (40%) seropositive
			Horses	63	28 (44%) seropositive
			Pigs	161	37 (23%) seropositive
[171]	Tokelau	1998	Pigs	88	3 (3.4%) seropositive

## Discussion

This systematic review is the first to synthesize and compile data on the epidemiology of human leptospirosis and pathogenic *Leptospira* spp. infection in animals in the PIs. Considering the limited population and economic resources in those mostly small and isolated islands, the number of studies retrieved was impressive (n = 148). Overall, the systematic review demonstrates that leptospirosis is an important cause of acute febrile illness across the PIs, with evidence of human disease demonstrated in 13 of 14 PIs where investigations have been conducted. Community-based seroprevalence studies were conducted in seven PIs and provided heterogeneous results, with prevalence ranging from 10% to 88%. Taken together, these findings reflect the public health importance of leptospirosis in the region, and corroborate recent estimates of very high disease burden in Oceania [3]. A wide range of domestic and wildlife species (n = 13 when counting rats as a single species) from across the PIs showed evidence of present and/or past infection with *Leptospira*. In some PIs, infected animals were mainly livestock (cattle and pigs in particular), indicating the potentially important role of non-rodent reservoir species in human infections, especially in islands where backyard subsistence livestock are common. However, rats were investigated in fewer studies and less islands than livestock, even in islands where serogroup Icterohaemorrhagiae was identified from human cases (e.g. Palau).

Even though many studies on human leptospirosis in the PIs were retrieved, these studies probably under-estimate the true burden as leptospirosis is thought to be under-reported globally, particularly in developing countries such as the majority of PIs. Several studies included in our review were conducted during outbreaks of dengue [81, 142, 150], chikungunya [24] or murine typhus [76], and coincidentally identified leptospirosis cases that might otherwise have remained undetected. On the contrary, the co-circulation of different infectious diseases sharing similar clinical presentation in the PIs might lead to leptospirosis under-diagnosis, sometimes resulting in a fatal outcome [24]. In the reviewed human clinical studies, diagnosis for leptospirosis often occurred very late, in already hospitalised (n = 21 studies where hospitalisation is specified) or even deceased patients [164]. A case report from American Samoa highlighted some of the leptospirosis diagnostic challenges faced by clinicians in the PIs [143]. There is still a need to raise awareness and to reinforce diagnostic capabilities for leptospirosis in the PIs. These have to go hand in hand with better agreement about the use of diagnostic tools and interpretation of the results. The development of clinical guidelines for the management of febrile patients with suspected leptospirosis should be a priority.

Serological studies suggested a wide variety of *Leptospira* serogroups (n = 21) in humans and animals across the PIs, including common as well as rare serogroups. Reported serogroups varied between PIs, between humans and animals, and between animal species. Across the PIs, seropositive animal hosts were reported for all the serogroups detected in human cases. However, it was difficult to make serogroup links between animals and humans because data were reported from multiple studies with variable study designs. Also, MAT results were difficult to interpret because of the intrinsic limitations of the test, as well as the heterogeneity in data quality between the studies. MAT has limited sensitivity, especially with early acute-phase specimens, and with chronically infected animals (reservoirs) that may be serologically negative even while shedding the bacteria in their urine [173]. Also, MAT results may vary depending on subjective interpretation by laboratory personnel and the panel of serovars used [2]. Interpretation is further complicated by the high degree of cross-reactions that occurs between *Leptospira* of the same serogroup or even between serogroups [173]. Apart from those intrinsic limitations, many studies included in our review were classified as “weak evidence” because they did not comply with our case definition and/or provide the minimal information required to assess the quality of the results. Even in studies complying with our case definition, some MAT results were still overstated. For example, one study from New Caledonia [57] reported 12 different serogroups in severe cases identified in 1989, while studies using *Leptospira* isolates or genotyping techniques have provided evidence that only six serogroups have been responsible for human cases in New Caledonia [60, 98]. Therefore, tables of serology results provided in this review (Table 1 and S2 Table) should be taken as summaries of the reports from the studies, but interpreted with great caution. To ensure a proper quality assessment of the results and accurate interpretation, it is crucial that in the future MAT results are reported in detail, especially providing information regarding the panel of serovars used, MAT titres, and detailed and appropriate case definition criteria. In cases where serology is expected to be of poor sensitivity, the use of molecular methods might be more appropriate. In our review, a limited number of studies relied on a molecular diagnosis (n = 14 in animals; n = 15 in humans).

Studies including both animal and clinical data were uncommon (n = 13 in 11 PIs) in this review. Also, molecular characterization of *Leptospira* spp. diversity that allows human *Leptospira* infection to be traced back to the probable source of contamination [172] was attempted in very few studies. When *Leptospira* infection was investigated in separate studies for animals and humans in a same PI, it was generally not possible to link the results, for example because of a time gap between animal and human studies, and/or different study designs, and/or the use of different MAT panels. In some PIs, human leptospirosis was not investigated, even though *Leptospira* infection was demonstrated in animals, i.e. in the Solomon Islands, Cook Islands, Kiribati, Niue, Tokelau, Samoa, and to a lesser extent Tonga where only four suspect human cases were tested. Also, we retrieved livestock studies from 19 PIs while rodent studies were available from only eight of these 19 PIs, which might bias our understanding of leptospirosis infection in humans in the PIs.

This review identified common behavioural risk factors and environmental drivers for leptospirosis infection across the region. Environmental drivers were mostly climate-related (flooding, extreme events), while individual risk factors included backyard subsistence livestock, farming, lifestyle (walking bare-foot, fishing, hunting), contact with animals and contact with fresh water sources (recreational use, washing laundry). Our review only identified a few epidemiological surveys that conducted robust statistical analyses of risk factors associated with human leptospirosis. Many studies reported some “possible drivers” of infection, or some “possible exposure” but the hypotheses were rarely properly evaluated. Even where surveys on clinical cases were conducted with detailed questionnaires, the probable source of infection was difficult to ascertain because of the multiplicity of potential infecting exposures (e.g. people who have been in contact with rats and pigs and dogs, and have been swimming in fresh water), the overlapping of professional and recreational activities and the lack of a comparator or control group.

Leptospirosis as a human disease is the result of complex interactions between humans, animal reservoirs/carriers, and the environment where the bacteria can survive. Our findings suggest that the major animal reservoirs of human-infecting leptospires may vary across the PIs, but that livestock (especially cattle and pigs), dogs and rodents may all play important roles in disease transmission to humans. However, intra-island heterogeneity has not been explored. In Fiji, the difference between habitats has been specifically investigated, showing that the relative importance of animal species in human infections varied between urban, peri-urban and rural settings [111]. We also showed a wide heterogeneity of leptospirosis serogroups and individual risk factors within and between islands. Combined with the already discussed limitations of the MAT and the limited number of studies combining animal, human and environmental investigations, overall the epidemiology of the disease is still unclear for most of the PIs.

As already advocated in other studies [174, 175], eco-epidemiological studies following an integrated “One Health” approach are needed to understand the exposure pathways for leptospirosis in humans, including the specific role and relative importance of each animal species in different environmental settings. Future studies should further explore how disease transmission to humans is influenced by the complex interactions between humans, animals, and the environment, including interactions within as well as between ecological scales. Also, better integration of environmental studies (only two in our search), inclusion of health economics evaluations and application of novel epidemiological methods such as mathematical modelling would be valuable next steps. This is crucial in a context where climate change, increased risk of flooding, population growth, urbanization, loss of biodiversity and agricultural intensification could individually, or possibly synergistically, lead to further increases in the burden of human leptospirosis in the Pacific. In the future, guidelines for a proper framework for leptospirosis research may help to improve our understanding of local epidemiology and complex transmission dynamics of leptospirosis worldwide.

## Supporting information

S1 Appendix PRISMA checklist. (DOC)

S2 Appendix Search strategy. A list of electronic databases browsed is provided, as well as the complete search strategy, including search terms.
(DOC)

S1 Table List of included studies. The complete reference is provided for each study, as well as the island(s) where the study took place, the target (human *vs* animal) and the type of study. Human clinical studies were reported as strong or weak evidence of leptospirosis following the criteria detailed in the methods section. The 11 studies for which the full text document could not be retrieved are indicated.
(XLSX)

S2 Table Detailed serology results. The results are reported per study, indicating where (island(s)) and when (year(s)) the study was conducted, the target (human *vs* animal), the animal species investigated when relevant and the serogroup. Serology results are reported as in the study, i.e. potentially including cross-reactions between serovars and serogroups, paradoxical reactions or anamnestic responses. For this reason, and because of the limitations of the MAT, some results were considered as “weak evidence” of leptospirosis following the criteria detailed in the methods section; thus this table should be interpreted with caution.
(XLS)
