# Supplementary material for: A systematic review of human and animal leptospirosis in the Pacific Islands reveals pathogen and reservoir diversity
Source: PLoS Negl Trop Dis. 2018 May 14;12(5):e0006503. doi: 10.1371/journal.pntd.0006503 (PMC5967813; doi:10.1371/journal.pntd.0006503)
Supplement: S2 Appendix — A list of electronic databases browsed is provided, as well as the complete search strategy, including search terms. (DOC) [file pntd.0006503.s002.doc]

**S2 Appendix**

**Electronic databases**

The following list includes electronic databases that were assessed for the systematic review:

| **Database** | **Website** |
| --- | --- |
| ***Global databases***  - PubMed  - Embase  - Web of Science  - Scopus  ***Websites browsed for grey literature***  - Google Scholar  - DASS de Nouvelle Calédonie    - Secretariat of the Pacific Community Public Health Division  - Pacific Health Dialog and Pacific Health Voices  - World Health Organisation, Western Pacific Region  - PNG Department of Health  - Government of Guam  - Samoa Ministry of Health  - Government of Tonga Ministry of Health  - Republic of Palau National Government Ministry of Health | <https://www.ncbi.nlm.nih.gov/pubmed/>  https://www.embase.com/  <https://webofknowledge.com/>  https://www.scopus.com/search/form.uri  <https://scholar.google.com/>  <http://www.dass.gouv.nc/portal/page/portal/dass/observatoire_sante/veille_sanitaire/Leptospirose>  <http://www.spc.int/php/>  <http://www.pacifichealthvoices.org/>  <http://www.wpro.who.int/en/>  <http://www.health.gov.pg/>  <http://www.guam.gov/>  <http://www.health.gov.ws/>  <http://www.health.gov.to/>  <http://www.palauhealth.org/> |

**Search strategy (four international databases)**

- The PubMed search was as follows:

**Search 1:** Search (((Oceania*[TIAB] OR "Oceania"[TIAB] OR "Australasia"[TIAB] OR "Melanesia"[TIAB] OR "Fiji"[TIAB] OR "New Caledonia"[TIAB] OR "Papua New Guinea"[TIAB] OR "Papua and New Guinea"[TIAB] OR "Vanuatu"[TIAB] OR "Micronesia"[TIAB] OR "Guam"[TIAB] OR "Palau"[TIAB] OR "Polynesia"[TIAB] OR "Hawaii"[TIAB] OR "Pitcairn Island"[TIAB] OR "Samoa"[TIAB] OR "American Samoa”[TIAB] OR "Independent State of Samoa”[TIAB] OR "Tonga"[ TIAB] OR "easter island”[TIAB] OR "french polynesia”[TIAB] OR "niue”[TIAB] OR "tokelau”[TIAB] OR "tokelau island*”[TIAB] OR "wake island”[TIAB] OR "wallis island”[TIAB] OR "wallis”[TIAB] OR "futuna”[TIAB] OR "wallis and futuna”[TIAB] OR "cook islands”[TIAB] OR "tahiti”[TIAB] OR "norfolk island"[Tiab] OR OR "new hebrides"[Tiab] OR "solomon islands”[TIAB] OR "johnston island”[TIAB] OR "kiribati”[TIAB] OR "gilbert islands”[TIAB] OR "mariana islands”[TIAB] OR "marshall islands”[TIAB] OR "nauru”[TIAB] OR "tuvalu”[TIAB] OR "ellice islands”[TIAB] OR "caroline islands”[TIAB] OR "marquises”[TIAB] OR "marquesas”[TIAB])) OR (Pacific[TIAB] AND island*[TIAB])) OR ("Oceania"[Mesh] OR "Australasia"[Mesh] OR "Melanesia"[Mesh] OR "Fiji"[Mesh] OR "New Caledonia"[Mesh] OR "Papua New Guinea"[Mesh] OR "Papua and New Guinea"[Mesh] OR "Vanuatu"[Mesh] OR "Micronesia"[Mesh] OR "Guam"[Mesh] OR "Palau"[Mesh] OR "Polynesia"[Mesh] OR "Hawaii"[Mesh] OR "Pitcairn Island"[Mesh] OR "Samoa"[Mesh] OR "American Samoa"[Mesh] OR "Independent State of Samoa"[Mesh] OR "Tonga"[Mesh])

**Search 2:** Search ((Leptospir*[tw]) OR Leptospir*[tiab]) OR ("Leptospirosis"[Mesh] OR "Leptospiraceae"[Mesh] OR "Leptospira"[Mesh])

**Search 3:** combine search 1 AND search 2

- For Web of Science, we queried:

Search box 1: Topic=Leptospir* AND search box 2: Topic=Oceania*

OR

Search box 1: Topic=Leptospir* AND search box 2: Topic=Pacific Islands

OR

Search box 1: Topic=Leptospir* AND search box 2: Topic=Polynesia*

- The Embase search was as follows:

**Search 1:** 'leptospirosis':ab,fx,hw,kw,ot,ti,tw OR 'leptospira': ab,fx,hw,kw,ot,ti,tw OR 'leptospiral': ab,fx,hw,kw,ot,ti,tw NOT [medline]/lim

**Search 2:** 'oceania':mp OR 'australasia':mp OR 'melanesia':mp OR 'micronesia':mp OR 'fiji':mp OR 'new caledonia':mp OR 'vanuatu':mp OR 'guam':mp OR 'palau':mp OR 'polynesia':mp OR 'hawaii':mp OR 'pitcairn island':mp OR 'samoa':mp OR 'american samoa':mp OR 'tonga':mp OR 'pacific island':mp OR 'pacific islands':mp OR 'easter island':mp OR 'french polynesia':mp OR 'niue':mp OR 'tokelau':mp OR 'wake island':mp OR 'wallis':mp OR 'futuna':mp OR 'wallis and futuna':mp OR 'cook islands':mp OR 'tahiti':mp OR 'norfolk island':mp OR 'new hebrides': :mp OR 'solomon islands':mp OR 'kiribati':mp OR 'mariana islands':mp OR 'marshall islands':mp OR 'nauru':mp OR 'tuvalu':mp OR 'ellice islands':mp OR 'caroline islands':mp OR 'papua and new guinea':mp OR 'papua new guinea':mp OR 'marquises':mp OR 'marquesas':mp [mp=title, abstract, heading word, original title, device manufacturer, drug manufacturer, device trade name, keyword, floating subheading word]

**Search 3:** combine #1 AND #2

Refined by type of reference :

#3 AND ('article'/it OR 'article in press'/it OR 'conference abstract'/it OR 'conference paper'/it OR 'letter'/it)

- The SCOPUS search was as follows:

Advanced search:

TITLE-ABS-KEY ( leptospir* ) AND SUBJAREA ( mult OR agri OR bioc OR immu OR neur OR phar OR mult OR medi OR nurs OR vete OR dent OR heal )

Refine results:

AND ( EXCLUDE ( DOCTYPE , "re" ) OR EXCLUDE ( DOCTYPE , "ed" ) OR EXCLUDE ( DOCTYPE , "le" ) )

AND ( LIMIT-TO ( AFFILCOUNTRY , "New Caledonia" ) OR LIMIT-TO ( AFFILCOUNTRY , "French Polynesia" ) OR LIMIT-TO ( AFFILCOUNTRY , "Fiji" ) OR LIMIT-TO ( AFFILCOUNTRY , "Federated States of Micronesia" ) OR LIMIT-TO ( AFFILCOUNTRY , "Guam" ) OR LIMIT-TO ( AFFILCOUNTRY , "Vanuatu" ) OR LIMIT-TO ( AFFILCOUNTRY , "Wallis and Futuna" ) )

AND ( EXCLUDE ( AFFILCOUNTRY , "New Zealand" ) OR EXCLUDE ( AFFILCOUNTRY , "United States" ) OR EXCLUDE ( AFFILCOUNTRY , "Brazil" ) OR EXCLUDE ( AFFILCOUNTRY , "India" ) OR EXCLUDE ( AFFILCOUNTRY , "France" ) OR EXCLUDE ( AFFILCOUNTRY , "United Kingdom" ) OR EXCLUDE ( AFFILCOUNTRY , "Australia" ) OR EXCLUDE ( AFFILCOUNTRY , "Italy" ) OR EXCLUDE ( AFFILCOUNTRY , "Germany" ) OR EXCLUDE ( AFFILCOUNTRY , "Japan" ) OR EXCLUDE ( AFFILCOUNTRY , "China" ) OR EXCLUDE ( AFFILCOUNTRY , "Netherlands" ) OR EXCLUDE ( AFFILCOUNTRY , "Thailand" ) OR EXCLUDE ( AFFILCOUNTRY , "New Zealand" ) OR EXCLUDE ( AFFILCOUNTRY , "Canada" ) OR EXCLUDE ( AFFILCOUNTRY , "Switzerland" ) OR EXCLUDE ( AFFILCOUNTRY , "Argentina" ) OR EXCLUDE ( AFFILCOUNTRY , "Spain" ) OR EXCLUDE ( AFFILCOUNTRY , "Iran" ) OR EXCLUDE ( AFFILCOUNTRY , "Malaysia" ) OR EXCLUDE ( AFFILCOUNTRY , "Taiwan" ) OR EXCLUDE ( AFFILCOUNTRY , "Turkey" ) OR EXCLUDE ( AFFILCOUNTRY , "Cuba" ) OR EXCLUDE ( AFFILCOUNTRY , "Mexico" ) OR EXCLUDE ( AFFILCOUNTRY , "Colombia" ) OR EXCLUDE ( AFFILCOUNTRY , "Sri Lanka" ) OR EXCLUDE ( AFFILCOUNTRY , "Belgium" ) OR EXCLUDE ( AFFILCOUNTRY , "Chile" ) OR EXCLUDE ( AFFILCOUNTRY , "Poland" ) OR EXCLUDE ( AFFILCOUNTRY , "South Korea" ) OR EXCLUDE ( AFFILCOUNTRY , "Israel" ) OR EXCLUDE ( AFFILCOUNTRY , "Ireland" ) OR EXCLUDE ( AFFILCOUNTRY , "Sweden" ) OR EXCLUDE ( AFFILCOUNTRY , "Greece" ) OR EXCLUDE ( AFFILCOUNTRY , "Russia" ) OR EXCLUDE ( AFFILCOUNTRY , "South Africa" ) OR EXCLUDE ( AFFILCOUNTRY , "Philippines" ) OR EXCLUDE ( AFFILCOUNTRY , "Denmark" ) OR EXCLUDE ( AFFILCOUNTRY , "Peru" ) OR EXCLUDE ( AFFILCOUNTRY , "Austria" ) OR EXCLUDE ( AFFILCOUNTRY , "Barbados" ) OR EXCLUDE ( AFFILCOUNTRY , "Croatia" ) OR EXCLUDE ( AFFILCOUNTRY , "Bulgaria" ) OR EXCLUDE ( AFFILCOUNTRY , "Czech Republic" ) OR EXCLUDE ( AFFILCOUNTRY , "Hungary" ) OR EXCLUDE ( AFFILCOUNTRY , "Romania" ) OR EXCLUDE ( AFFILCOUNTRY , "Indonesia" ) OR EXCLUDE ( AFFILCOUNTRY , "Tanzania" ) OR EXCLUDE ( AFFILCOUNTRY , "Czechoslovakia" ) OR EXCLUDE ( AFFILCOUNTRY , "Germany (Democratic Republic, DDR)" ) OR EXCLUDE ( AFFILCOUNTRY , "Portugal" ) OR EXCLUDE ( AFFILCOUNTRY , "Hong Kong" ) OR EXCLUDE ( AFFILCOUNTRY , "Egypt" ) OR EXCLUDE ( AFFILCOUNTRY , "Russian Federation" ) OR EXCLUDE ( AFFILCOUNTRY , "Slovakia" ) OR EXCLUDE ( AFFILCOUNTRY , "Singapore" ) OR EXCLUDE ( AFFILCOUNTRY , "Trinidad and Tobago" ) OR EXCLUDE ( AFFILCOUNTRY , "Laos" ) OR EXCLUDE ( AFFILCOUNTRY , "Venezuela" ) OR EXCLUDE ( AFFILCOUNTRY , "Morocco" ) OR EXCLUDE ( AFFILCOUNTRY , "Norway" ) OR EXCLUDE ( AFFILCOUNTRY , "Finland" ) OR EXCLUDE ( AFFILCOUNTRY , "Viet Nam" ) OR EXCLUDE ( AFFILCOUNTRY , "Yugoslavia" ) OR EXCLUDE ( AFFILCOUNTRY , "Nigeria" ) OR EXCLUDE ( AFFILCOUNTRY , "Jamaica" ) OR EXCLUDE ( AFFILCOUNTRY , "Ecuador" ) OR EXCLUDE ( AFFILCOUNTRY , "Pakistan" ) OR EXCLUDE ( AFFILCOUNTRY , "Puerto Rico" ) OR EXCLUDE ( AFFILCOUNTRY , "Cambodia" ) OR EXCLUDE ( AFFILCOUNTRY , "Kenya" ) OR EXCLUDE ( AFFILCOUNTRY , "Nepal" ) OR EXCLUDE ( AFFILCOUNTRY , "Uruguay" ) OR EXCLUDE ( AFFILCOUNTRY , "Zimbabwe" ) OR EXCLUDE ( AFFILCOUNTRY , "Bangladesh" ) OR EXCLUDE ( AFFILCOUNTRY , "Saudi Arabia" ) OR EXCLUDE ( AFFILCOUNTRY , "Slovenia" ) OR EXCLUDE ( AFFILCOUNTRY , "Sudan" ) OR EXCLUDE ( AFFILCOUNTRY , "Nicaragua" ) OR EXCLUDE ( AFFILCOUNTRY , "Serbia" ) OR EXCLUDE ( AFFILCOUNTRY , "Uganda" ) OR EXCLUDE ( AFFILCOUNTRY , "Bolivia" ) OR EXCLUDE ( AFFILCOUNTRY , "Costa Rica" ) OR EXCLUDE ( AFFILCOUNTRY , "Guadeloupe" ) OR EXCLUDE ( AFFILCOUNTRY , "Reunion" ) OR EXCLUDE ( AFFILCOUNTRY , "Senegal" ) OR EXCLUDE ( AFFILCOUNTRY , "Seychelles" ) OR EXCLUDE ( AFFILCOUNTRY , "United Arab Emirates" ) OR EXCLUDE ( AFFILCOUNTRY , "Madagascar" ) OR EXCLUDE ( AFFILCOUNTRY , "Ukraine" ) OR EXCLUDE ( AFFILCOUNTRY , "Albania" ) OR EXCLUDE ( AFFILCOUNTRY , "Armenia" ) OR EXCLUDE ( AFFILCOUNTRY , "Gabon" ) OR EXCLUDE ( AFFILCOUNTRY , "Kazakhstan" ) OR EXCLUDE ( AFFILCOUNTRY , "Lithuania" ) OR EXCLUDE ( AFFILCOUNTRY , "Martinique" ) OR EXCLUDE ( AFFILCOUNTRY , "Bosnia and Herzegovina" ) OR EXCLUDE ( AFFILCOUNTRY , "Cameroon" ) OR EXCLUDE ( AFFILCOUNTRY , "Ethiopia" ) OR EXCLUDE ( AFFILCOUNTRY , "Georgia" ) OR EXCLUDE ( AFFILCOUNTRY , "Ghana" ) OR EXCLUDE ( AFFILCOUNTRY , "Grenada" ) OR EXCLUDE ( AFFILCOUNTRY , "Lebanon" ) OR EXCLUDE ( AFFILCOUNTRY , "Luxembourg" ) OR EXCLUDE ( AFFILCOUNTRY , "Saint Kitts and Nevis" ) OR EXCLUDE ( AFFILCOUNTRY , "Bhutan" ) OR EXCLUDE ( AFFILCOUNTRY , "Botswana" ) OR EXCLUDE ( AFFILCOUNTRY , "Congo" ) OR EXCLUDE ( AFFILCOUNTRY , "Guyana" ) OR EXCLUDE ( AFFILCOUNTRY , "Mozambique" ) OR EXCLUDE ( AFFILCOUNTRY , "Namibia" ) OR EXCLUDE ( AFFILCOUNTRY , "Niger" ) OR EXCLUDE ( AFFILCOUNTRY , "Panama" ) OR EXCLUDE ( AFFILCOUNTRY , "Suriname" ) OR EXCLUDE ( AFFILCOUNTRY , "Tunisia" ) OR EXCLUDE ( AFFILCOUNTRY , "Cyprus" ) OR EXCLUDE ( AFFILCOUNTRY , "Dominican Republic" ) OR EXCLUDE ( AFFILCOUNTRY , "French Guiana" ) OR EXCLUDE ( AFFILCOUNTRY , "Jordan" ) OR EXCLUDE ( AFFILCOUNTRY , "Kuwait" ) OR EXCLUDE ( AFFILCOUNTRY , "Malta" ) OR EXCLUDE ( AFFILCOUNTRY , "Netherlands Antilles" ) OR EXCLUDE ( AFFILCOUNTRY , "Oman" ) OR EXCLUDE ( AFFILCOUNTRY , "Paraguay" ) OR EXCLUDE ( AFFILCOUNTRY , "Zambia" ) OR EXCLUDE ( AFFILCOUNTRY , "Algeria" ) OR EXCLUDE ( AFFILCOUNTRY , "Andorra" ) OR EXCLUDE ( AFFILCOUNTRY , "Angola" ) OR EXCLUDE ( AFFILCOUNTRY , "Aruba" ) OR EXCLUDE ( AFFILCOUNTRY , "Belize" ) OR EXCLUDE ( AFFILCOUNTRY , "Brunei Darussalam" ) OR EXCLUDE ( AFFILCOUNTRY , "Cape Verde" ) OR EXCLUDE ( AFFILCOUNTRY , "Cayman Islands" ) OR EXCLUDE ( AFFILCOUNTRY , "Cote d'Ivoire" ) OR EXCLUDE ( AFFILCOUNTRY , "Dominica" ) OR EXCLUDE ( AFFILCOUNTRY , "Estonia" ) OR EXCLUDE ( AFFILCOUNTRY , "Falkland Islands (Malvinas)" ) OR EXCLUDE ( AFFILCOUNTRY , "Faroe Islands" ) OR EXCLUDE ( AFFILCOUNTRY , "Greenland" ) OR EXCLUDE ( AFFILCOUNTRY , "Guatemala" ) OR EXCLUDE ( AFFILCOUNTRY , "Latvia" ) OR EXCLUDE ( AFFILCOUNTRY , "Libyan Arab Jamahiriya" ) OR EXCLUDE ( AFFILCOUNTRY , "Mali" ) OR EXCLUDE ( AFFILCOUNTRY , "Mauritius" ) OR EXCLUDE ( AFFILCOUNTRY , "Mayotte" ) OR EXCLUDE ( AFFILCOUNTRY , "Mongolia" ) OR EXCLUDE ( AFFILCOUNTRY , "North Korea" ) OR EXCLUDE ( AFFILCOUNTRY , "Palestine" ) OR EXCLUDE ( AFFILCOUNTRY , "Saint Helena" ) OR EXCLUDE ( AFFILCOUNTRY , "Saint Pierre and Miquelon" ) OR EXCLUDE ( AFFILCOUNTRY , "Sierra Leone" ) ) AND ( EXCLUDE ( DOCTYPE , "re" ) OR EXCLUDE ( DOCTYPE , "le" ) OR EXCLUDE ( DOCTYPE , "ed" ) )

AND ( LIMIT-TO ( LANGUAGE , "English" ) OR LIMIT-TO ( LANGUAGE , "French" ))
